# Physiological and Structural Responses to Prolonged Water Deficit in Young Trees of Two Olive Cultivars

**DOI:** 10.3390/plants11131695

**Published:** 2022-06-27

**Authors:** Roberto Massenti, Alessio Scalisi, Francesco Paolo Marra, Tiziano Caruso, Giulia Marino, Riccardo Lo Bianco

**Affiliations:** 1 Department of Agricultural, Food and Forest Sciences (SAAF), University of Palermo, 90128 Palermo, Italy; alessio.scalisi@agriculture.vic.gov.au (A.S.); francescopaolo.marra@unipa.it (F.P.M.); tiziano.caruso@unipa.it (T.C.); giumarino@ucanr.edu (G.M.); riccardo.lobianco@unipa.it (R.L.B.); 2 Tatura SmartFarm, Agriculture Victoria, Tatura, VIC 3616, Australia; 3 Department of Plant Sciences, University of California, Davis, CA 95616, USA

**Keywords:** conductance, drought, leaf turgor, *Olea europaea* L., photosynthesis, transpiration, trunk diameter, water stress

## Abstract

This study aimed to characterize the physiological and structural responses of potted one-year-old olive trees belonging to two olive cultivars—‘Nocellara del Belice’ and ‘Cerasuola’—exposed to prolonged drought under greenhouse conditions. Two irrigation treatments based on evapotranspiration (ET) were imposed for 69 days, i.e., well-watered (WW, 100% ET) and drought-stressed (DS, 10–30% ET). Leaf stomatal conductance (g_s_), stem water potential (Ψ_stem_), transpiration (E), photosynthetic capacity (A_max_), water use efficiency (WUE), stem (K_stem_) and root (K_root_) hydraulic conductance, trunk diameter variations (TDV), and leaf patch attenuated pressure fluctuations (p_p_, a proxy of the inverse of leaf turgor pressure) were measured in WW and DS trees at different stages of the experiment. Leaf g_s_ did not significantly differ between cultivars under DS, whereas differences in Ψ_stem_ only became significant at the end of prolonged drought, when ‘Nocellara del Belice’ experienced Ψ_stem_ < −4 MPa. ‘Cerasuola’ trees expressed the best WUE under drought, although they were more susceptible to photoinhibition under optimal plant water status. Both cultivars tended to increase their K_stem_ at the end of the drought period. A marked reduction in K_root_ occurred in ‘Cerasuola’ plants after prolonged drought; however, a similar mechanism was not observed in ‘Nocellara del Belice’. The ratio between K_stem_ and K_root_ exponentially increased towards the end of the prolonged drought period in both cultivars, but more markedly in ‘Cerasuola’. TDV and p_p_ trends suggested that ‘Cerasuola’ plants keep better plant water status under severe drought compared to ‘Nocellara del Belice’ by maintaining high leaf turgor and reduced trunk diameter fluctuations. These responses may be related to reduced cell wall elasticity and xylem vessel size and/or wall thickness—drought avoidance mechanisms. The K_stem_/K_root_ ratio can serve as an indicator of drought stress avoidance mechanisms to compare genotype-specific responses to drought stress.

## 1. Introduction

In recent years, climate change has led to a reconsideration of the approaches to irrigation practices in agriculture, as its effects in the Mediterranean countries may cause alarming scenarios [1,2]. Factors such as more sporadic but intense precipitations and increased temperatures leading to more frequent summer heatwaves affect the incidence of abiotic stresses on tree crops (e.g., limiting or excessive water, heat, light availability).

In olive growing, maintaining production and unchanged quality standards is a priority. Many strategies, based on plant physiological responses to water deficit, can be used to maintain the plants in a range of low water stress. However, current irrigation management is based on protocols mainly related to phenological stages (e.g., regulated deficit irrigation) and often rely on visual observations of foliage stress symptoms such as wilting and adaxial leaf movement to increase radiation reflection [3]. On larger scales, farmers use sensors to monitor soil moisture and measure weather variables to manage irrigation more precisely. Plant-based sensors are still relatively uncommon in commercial farms. Nevertheless, early and reliable plant stress indicators are needed to avoid permanent stress effects. Often, mild water stress is a valuable tool for the natural control of vigor, and it may be used to improve the quality of fruit or derived products (e.g., olive oil).

New olive planting systems are increasingly focused on minimizing the unproductive period and increasing yields, instead of favoring constant productivity and the efficient mechanization of harvesting [4]. It has been demonstrated that proper irrigation management can reduce tree vegetative growth without altering productive efficiency [5,6,7,8,9]. Excessive irrigation not only results in significant environmental damage due to water wastage but can also lead to increased plant vulnerability to abiotic and biotic stresses and, in the case of olive, to a reduction in the phenolic compounds in the oil [10].

Several studies have provided evidence of osmoregulation as a stress avoidance mechanism in olive [11,12,13,14,15,16]. Olive trees express a high capacity for osmotic adjustments under conditions of soil water shortage, and this allows the tree to reduce changes in cell turgor caused by decreasing leaf water potential [11,17]. Olive trees resist drought stress by lowering the water content and water potential of their tissues. This allows plants to establish a high potential gradient between leaves and roots, and thus extract soil water down to −2.5 MPa [13]. Accumulation of some sugars occurs in olive leaf and root cells [14,16,18], particularly mannitol, a sugar alcohol that plays a very important role for cell osmotic adjustment. In highly stressed olive trees, the cellular concentration of mannitol increases by 97%, and, combined with glucose, they cause a reduction of 0.32 MPa in total osmotic potential [14].

Long periods of severe water deficit may lead to stomatal closure [19], inhibition of photosynthesis [20], reduced gas exchange [21], osmotic adjustments [22] mainly due to the accumulation of glucose and mannitol [16], over-regulation of some antioxidant enzymes [23], changes in root system growth [24], and morpho-anatomical adaptations of leaves such as reduced leaf area, and increased non-glandular leaf hairs and mesophyll cell density [25]. Severe dehydration predisposes the olive leaf chloroplast photosystems to photoinhibition due to a light-dependent deactivation of photochemical reactions [20]. Mannitol was thought to act as an oxygen radical scavenger in olive [26]. During the first days of the restoration of favorable water conditions following water deficit, olive plants only partially restore transpiration and photosynthetic processes, chlorophyll fluorescence indices, and osmotic potential [20,22]. This indicates that the rapid recovery of tissue water content is not accompanied by the recovery of leaf function, which may last for several days, depending on the level of stress reached earlier [20]. Under severe water stress, the effect of osmoregulation on leaf cell turgor was largely offset by xylem cavitation in ‘Meski’ and ‘Chemlali’ olives [27].

Fruit tree species that are more sensitive to water deficit than olive have a greater loss of hydraulic function due to cavitation, which also begins at lower stress levels [28]. In this regard, the olive tree hydraulic system manifests low vulnerability to cavitation events during periods of water deficit, and this is associated with low efficiency in xylem sap transport due to the fact that olive vessels have a small lumen and the transport of sap flows is reduced [28]. The low conductivity of olive trees is due to high hydraulic resistance in the stem and roots compared to other tree species. A combination of air vapor pressure deficit (VPD), genotype-specific hydraulic conductivity, and soil water potential determine the stem water potential in olive trees during transpiration. Hydraulic conductivity (K) in olive trees and in all fruiting tree species influences tree growth through changes in leaf water status and gas exchange [29,30].

Despite the fact that the literature shows evidence of olive cultivars deploying osmotic adjustments, reductions in cell wall elasticity, and other mechanisms of drought stress avoidance, only a few experiments have focused on comparisons among genotypes. Some olive genotypes have the ability to withstand water stress by decreasing the water content and water potential of their tissues, thereby establishing a strong gradient of water potential between leaves and roots [31] in order to draw water from the soil. Under severe water deficit conditions, olive plants limit canopy growth and reduce transpiration and photosynthetic processes [20,32]. This allows for the continuous production of assimilates and their accumulation in plant organs, such as in the root system, creating a higher root/leaf ratio than in well-watered plants [31].

The Sicilian olive germplasm is highly diverse, and it contains a pool of genotypes with different responses to water deficit [16,33,34,35]. Indeed, ‘Nocellara del Belice’ and ‘Cerasuola’ are characterized by their differing susceptibility to drought [36,37]. The hypothesis of this study was that the two cultivars may exhibit significantly different physiological and/or structural responses to prolonged water deficit. Specifically, this study aimed to investigate the responses of stem water potential, leaf stomatal conductance, transpiration, photosynthetic capacity, water use efficiency, stem and root hydraulic conductance, and trunk diameter and leaf turgor pressure variations in one-year-old trees of ‘Nocellara del Belice’ and ‘Cerasuola’ olive exposed to prolonged drought under greenhouse conditions.

## 2. Materials and Methods

### 2.1. Experimental Design

The experiment was carried out in 2017, in a greenhouse of the Department of Agricultural, Food and Forest Sciences at the University of Palermo (38°6′31.789′′ N and 13°21′2.271′′ E, 40 m a.s.l.). At the start of spring (end of March), 60 one-year-old self-rooted olive trees—30 ‘Nocellara del Belice’ (NB) and 30 ‘Cerasuola’ (CE) trees—were transplanted in 15-L pots using sandy loam soil. All the pots were irrigated to field capacity (FC) for two months, until 28 May, as trees acclimated to the greenhouse conditions. Afterwards, pots were split in two groups and subjected to two irrigation treatments. On 29 May, well-watered (WW) and drought-stressed (DS) irrigation treatments were imposed to 15 trees per each cultivar and irrigation combination. WW plants were irrigated for the entire experimental period to FC twice a week. The irrigation trial started on 29 May (day 0 of drought stress) and finished on 6 August (day 69 of drought stress). Evapotranspiration (ET) was determined by weighing WW pots before and after each irrigation event. The difference in weight between a pot after an irrigation event and the same pot before the following irrigation was equal to the total water lost by ET. The volume of water evapotranspired between irrigation events was reintegrated in WW pots at the next irrigation event—1 mL of water supplied for each gram of water lost by ET. The irrigation volume supplied to DS plants ranged between 30% and 10% of the ET of WW plants between two irrigation events. The 15 DS plants were exposed to different periods of water deficit. Five plants were exposed to 15 days of drought stress (DODS) with an irrigation equivalent of 30% ET of WW plants, five plants to 45 DODS also with 30% ET of WW plants, and five plants to the first 45 DODS with 30% ET of WW plants and the subsequent 24 DODS with 0–10% ET to induce higher water deficit in the final stages of drought exposure.

Temperature (T) and relative humidity (RH) were recorded at hourly intervals with a µMetos weather station (Pessl Instruments, Weiz, Austria) and used to calculate VPD. Weather data were then averaged in daily means.

### 2.2. Plant Water Status and Gas Exchange

#### 2.2.1. Leaf Stomatal Conductance

Leaf stomatal conductance (g_s_) was measured with a Delta-T AP4 dynamic porometer (Delta-T Devices LTD, Cambridge, UK). Mid-morning (10:00 to 11:00 HR, solar time) measurements of g_s_ (g_s-morning_) were carried out at weekly intervals on three leaves for each cultivar exposed to WW and DS treatments. In addition, g_s_ daily curves (i.e., from pre-dawn to evening) were obtained at 66 DODS to compare stomatal aperture responses under high water deficit against optimal irrigation.

#### 2.2.2. Stem Water Potential

A pressure chamber (PMS 600, Instrument Company, Albany, OR, USA) was used for the determination of stem water potential (Ψ_stem_) following the methodology adopted by Turner [38]. Midday Ψ_stem_ was measured at weekly intervals from the beginning of drought on three leaves for each cultivar exposed to WW and DS treatments. Similarly, daily curves of Ψ_stem_ were plotted using measurements carried out at 66 DODS from pre-dawn to 19:00 HR.

#### 2.2.3. Transpiration, Photosynthesis, and Water Use Efficiency

A CIRAS-3 portable photosynthesis system (PP Systems^®^, Hitchin, UK), equipped with a PLC3 universal leaf cuvette, was used to determine photosynthetic capacity (A_max_), transpiration (E), and water use efficiency (WUE) in response to increasing levels of photosynthetically active radiation (PAR), expressed as photosynthetic photon flux density (PPFD, 0–1600 µmol m^−2^ s^−1^). Measurements were conducted at 66 DODS and on three leaves per cultivar exposed to WW and DS treatments.

### 2.3. Trunk Cross-Sectional Area and Stem and Hydraulic Conductance

Trunk diameter was measured at weekly intervals, about 5 cm above the grafting point of all trees under trial, using a digital caliper and then converted in trunk cross-sectional area (TCSA).

At 15, 45, and 69 DODS, three trees for each cultivar and irrigation treatment were brought to the laboratory, removed from their pots, and above-ground and below-ground portions were dissected to determine hydraulic conductance (K, kg s^−1^ MPa^−1^) with a high-pressure flow meter (Gen-2, Dynamax Inc., Houston, TX, USA). We used the transient analysis method described by Tyree et al. [39] for K determination in stem and root sections. The flow was measured across an increasing water pressure gradient, and K data was subsequently normalized dividing it by the TCSA of the stem portion—an estimate of sapwood area in young trees—and was expressed as stem and root sapwood-specific conductance (K_stem_ and K_root_, respectively, kg s^−1^ cm^−2^ MPa^−1^).

### 2.4. Diel Dynamics of Trunk Diameter and Leaf Turgor Pressure

Trunk diameter variations (TDV) were continuously determined with fruit gauges adapted as stem dendrometers as described by Scalisi et al. [40]. The gauges were mounted on the stems of trees for six consecutive days and data were recorded with two CR1000 data loggers (Campbell Scientific Ltd., Leicestershire, UK) at 15-min intervals. Five days for each DS period were selected by removing the first day of each logging session to avoid an uncompleted 24-h dataset. Measurements were collected on six trees—three WW and three DS—at 39–43 DODS.

Leaf patch clamp pressure (LPCP) probes (Yara International, Oslo, Norway) were used to continuously monitor leaf turgor pressure variations in terms of attenuated pressure of leaf patches (p_p_), an index inversely related to leaf cell turgor pressure, as described by Zimmermann et al. [41]. LPCP probes were previously used to assess plant water status and responses to drought in olive genotypes [42,43]. These probes were clamped on the same days and trees used for TDV measurements.

Raw data from stem dendrometers and LPCP probes were smoothed using a 15-point convoluted spline function [44]. Subsequently, data were standardized using z-scores (i.e., z = (x − mean)/standard deviation) to compare and/or average data from trunks or leaves that had a different initial trunk diameter or leaf turgor pressure, respectively, when the sensors were clamped.

### 2.5. Statistical Analysis

The data were analyzed with two-way analysis of variance (repeated measures analysis of variance for TCSA trends over time) using cultivar and irrigation treatment as main factors, followed by Tukey’s post hoc test. Significant differences were reported using different letters (*p* < 0.05). Statistical analysis was performed using SYSTAT 13.1 (Systat software Inc., Chicago, IL, USA) procedures. Regression analysis on K_stem_/K_root_ data and graphs were plotted using SigmaPlot 12.5 (Systat software Inc., Chicago, IL, USA).

## 3. Results and Discussion

### 3.1. Greenhouse Environmental Conditions and Irrigation

**Figure 1:**
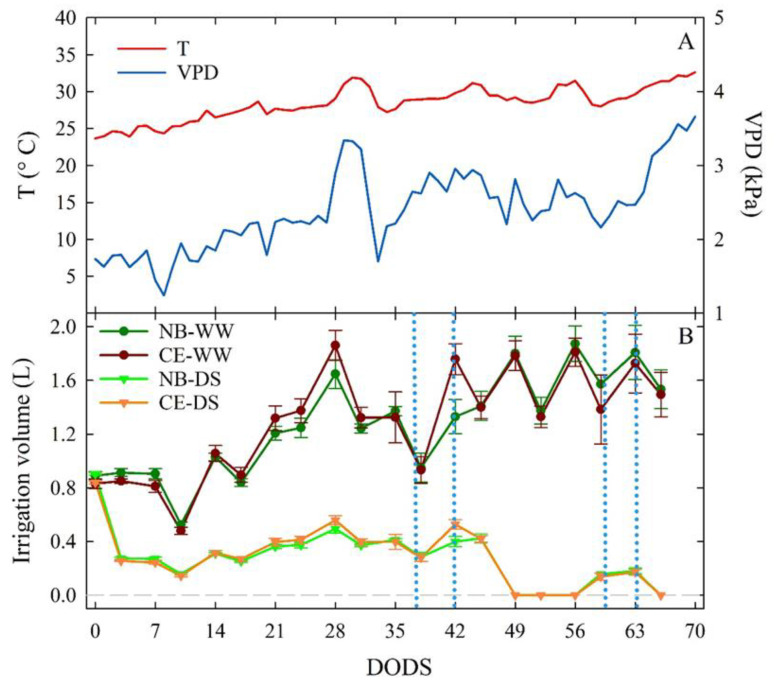
Daily trends of average temperature (T) and vapor pressure deficit (VPD) during the trial, expressed in days of drought stress (DODS) (**A**). Irrigation volumes supplied to well-watered and drought-stressed ‘Nocellara del Belice’ (NB-WW and NB-DS, respectively) and ‘Cerasuola’ (CE-WW and CE-DS, respectively) olive trees (**B**). Error bars in panel (**B**) indicate the standard errors of the means.

Average daily T values ranged between 24 and 34 °C from 0 to 70 DODS, with an increase at 28 DODS (end of June) and a decrease at 33 DODS (early July) (Figure 1A). T increased in the last seven days of the experiment and reached its maximum value on the last recorded day. VPD values fluctuated throughout the experiment, from 0.2 kPa, after the first week, to 3.7 kPa at the end of the trial. A peak of VPD was registered at 30 DODS, driven by a rise in T. As with T, a steady increase in VPD was seen in the last seven days of the trial (Figure 1A).

The fluctuation of irrigation volumes in NB-WW and CE-WW trees reflected the volumes of water evapotranspired between irrigation events. The highest volumes of water were supplied to trees in correspondence of high T and VPD due to increasing evapotranspiration. NB-DS and CE-DS olive trees received roughly similar volumes of water, only at 42 DODS did CE-DS receive slightly more water than NB-DS. In the last 20 DODS, water irrigation was reduced to 0–10% of ET to induce severe drought stress (Figure 1B).

### 3.2. Plant Water Status and Gas Exchange

#### 3.2.1. Leaf Stomatal Conductance and Stem Water Potential

**Figure 2:**
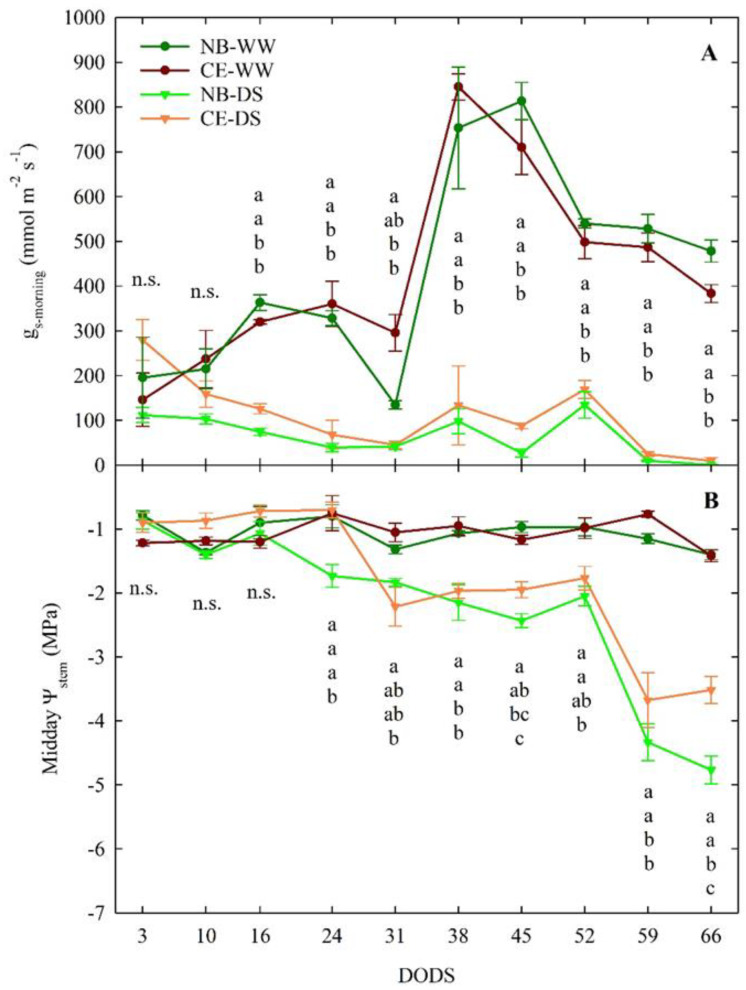
Mid−morning (1000 h) leaf stomatal conductance (g_s-morning_, (**A**)) and midday stem water potential (Ψ_stem_, (**B**)) of well-watered and drought-stressed ‘Nocellara del Belice’ (NB-WW and NB-DS, respectively) and ‘Cerasuola’ (CE-WW and CE-DS, respectively) olive trees. Error bars indicate the standard errors of the means. For each day of drought stress (DODS), different letters indicate significant differences among treatments according to Tukey’s test following analysis of variance (*p* < 0.05).

In the first 10 days from the start of the experiment, no significant g_s-morning_ differences (*p* > 0.05) were found among the four cultivar-irrigation combinations (Figure 2A). Leaf g_s-morning_ significantly decreased in DS compared to WW trees starting at 16 DODS, regardless of cultivar. These significant differences persisted until the end of the trial, with the only exception of 31 DODS, when, in response to high T and VPD, g_s-morning_ of NB-WW dropped and became not significantly different from NB-DS and CE-DS. A week later, g_s-morning_ in NB-WW and CE-WW soared, likely in response to lower values of T and VPD. Although measurements were taken at mid-morning, when maximum stomatal aperture is expected, at the end of the trial, DS trees expressed complete closure of stomata, as indicated by the near-zero values of g_s-morning_, that, in turn, might have led to significant reductions in photosynthetic activity and transpiration [45].

In WW plants of both cultivars, midday Ψ_stem_ was relatively constant, around a value of −1 MPa throughout the experimental period (Figure 2B). In the first 16 DODS, midday Ψ_stem_ did not show any differences between the two irrigation levels or cultivars, despite an irrigation supply of 30% ET in DS plants. Between 16 and 52 DODS, values of midday Ψ_stem_ in DS trees were between −1.5 and −2.5 MPa, which corresponds to mild drought stress in olive. CE-DS trees started to significantly reduce their midday Ψ_stem_ at 31 DODS, a week later than NB-DS. From 31 to 59 DODS, CE-DS maintained similar midday Ψ_stem_ to NB-DS, although the former often had slightly higher average values. From 52 to 66 DODS—when only 10% of ET was used to water DS trees—NB-DS and CE-DS showed a significant drop in midday Ψ_stem_. In this final part of the trial, NB-DS reached a minimum midday Ψ_stem_ value of −5.0 MPa, whereas the lowest value in CE-DS was −3.7 MPa. Despite the 10% ET irrigation, DS plants of both cultivars failed to reestablish adequate tissue hydration levels.

These results confirm that mildly stressed olive trees restrict excessive water loss and water potential drops by modulating stomatal closure [46], which is the earliest response to drought and the major limitation to photosynthesis at mild-moderate drought [47]. These findings also confirm that which has been previously observed in NB tress under deficit irrigation in high-density olive plantings, where NB expressed lower midday Ψ_stem_ than the Sicilian cultivar ‘Olivo di Mandanici’ [42,43]. This may be in part due to the inability of this cultivar to cope with drought stress only by promptly closing leaf stomata [16,37].

**Figure 3:**
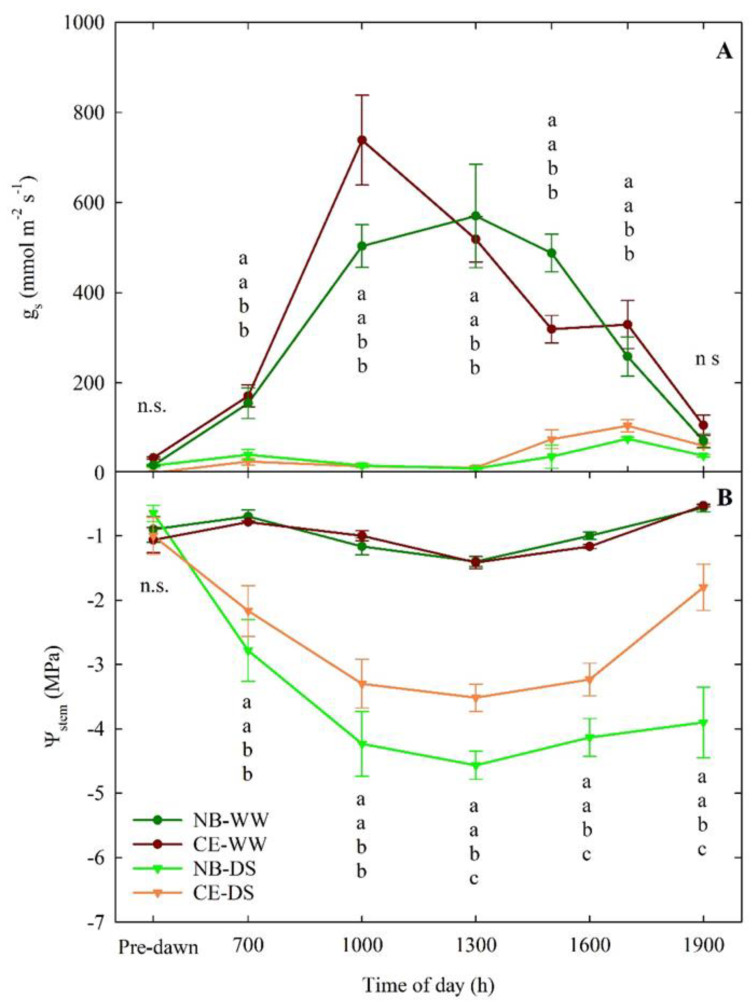
Daily−curves of leaf stomatal conductance (g_s_, (**A**)) and stem water potential (Ψ_stem_, (**B**)) of well-watered and drought-stressed ‘Nocellara del Belice’ (NB-WW and NB-DS, respectively) and ‘Cerasuola’ (CE-WW and CE-DS, respectively) olive trees. Measurements carried out on the 66th day of drought stress (DODS; 3 August 2017). Error bars indicate the standard errors of the means. For each day, different letters indicate significant differences among treatments according to Tukey’s test following analysis of variance (*p* < 0.05).

The daily patterns of g_s_ at the end of drought (66 DODS) showed markedly significant differences between WW and DS, regardless of the cultivar (Figure 3A). CE-WW plants showed an expected g_s_ peak at mid-morning, followed by a steady decrease in conductance until 1900 h. NB-WW did not have a clearly defined peak of g_s_, and its pattern followed a bell-like shape, with the highest g_s_ occurring between 1000 and 1500 h. Nevertheless, there were no significant differences between the NB-WW and CE-WW. Similarly, NB-DS and CE-DS did not significantly differ at any of the six measurement times (Figure 3A). In both NB-DS and CE-DS, g_s_ was near zero the entire day but there was a tendency to have slightly higher stomatal aperture in the afternoon (1500–1700 h), in line with previous observations in NB [42].

The daily patterns of Ψ_stem_ in WW treatments showed relatively smaller fluctuations than in DS trees (Figure 3B). No significant differences between the Ψ_stem_ of NB-WW and CE-WW were detected. Both NB-WW and CE-WW reached the lowest Ψ_stem_ of −1.4 Mpa at solar noon (i.e., 1300 h). In NB-DS and CE-DS, Ψ_stem_ was significantly lower than in NB-WW and CE-WW at all the measurement times except for pre-dawn measurements. NB-DS had significantly lower Ψ_stem_ than CE-DS from 1300 to 1900 h and reached its lowest value at 1300 h (−4.8 Mpa). CE-DS showed better Ψ_stem_ recovery at the end of the day, with values going back up to −1.8 Mpa, than NB-DS, which, at the same time, maintained a critically low value of −3.9 Mpa. Expectedly, WW plants of both cultivars showed a good ability to restore an optimal water status (−0.5 to −0.6 Mpa) at the end of the day. The low values of Ψ_stem_ observed in NB-DS are likely driven by other physiological mechanisms or genetic differences with CE. In a previous study, NB was classified as a leaf dehydration-intolerant cultivar, and leaf drop was one of the mechanisms triggered to avoid excessive dehydration [16].

#### 3.2.2. Transpiration, Photosynthesis, and Water Use Efficiency

**Figure 4:**
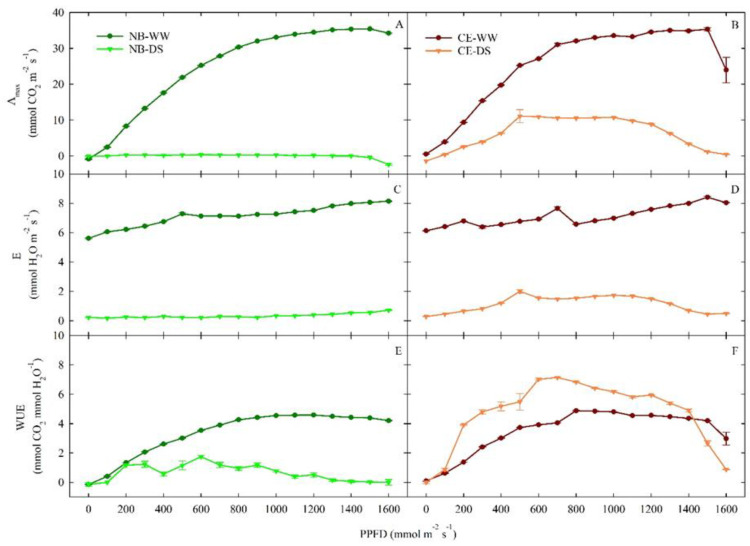
Responses of photosynthetic capacity (A_max_, (**A**,**B**)), transpiration (**C**–**E**), and water use efficiency (WUE, (**E**,**F**)) to changes in photosynthetic photon flux density (PPFD) in well-watered and drought-stressed ‘Nocellara del Belice’ (NB-WW and NB-DS, respectively) and ‘Cerasuola’ (CE-WW and CE-DS, respectively) olive trees on the 59th day of drought stress. Error bars indicate the standard errors of the means.

After nearly two months of drought stress (59 DODS), A_max_ showed similar responses to increasing PAR in leaves of NB-WW and CE-WW plants (Figure 4A,B). In both cultivars, when no water-limiting conditions were imposed, A_max_ reached a plateau (of ~35 µmol m^−2^ s^−1^) at PPFD ≥ 1200 µmol m^−2^ s^−1^. At the highest PPFD level (1600 µmol m^−2^ s^−1^), CE-WW leaves appeared to reduce their photosynthetic capacity, which suggests that CE may be slightly more susceptible to photoinhibition than NB. Photoinhibition may be pronounced in olive leaves exposed to high-PAR environments, and it interacts with water availability [48] by possibly genotype-dependent mechanisms, as shown by in ‘Biancolilla’ and ‘Coratina’ [49]. The E response pattern at increasing light exposure was similar in NB-WW and CE-WW (Figure 4C,D), and E steadily increased to values above 8 mmol m^−2^ s^−1^, which were reached at the maximum level of light imposed to leaves. On the other hand, the prolonged period of drought stress appeared to differently affect DS plants of the two cultivars. In NB-DS, A_max_ and E did not rise in response to increasing light availability and remained near zero (Figure 4A,C), suggesting complete closure of stomata in this cultivar under prolonged water scarcity. In CE-DS, A_max_ and E increased between 0 and 500 µmol m^−2^ s^−1^ PPFD, and then remained stable around 10.5 µmol CO_2_ m^−2^ s^−1^ and 1.7 mmol H_2_O m^−2^ s^−1^, respectively, under light exposure ranging from 500 to 1100 µmol m^−2^ s^−1^ PPFD. Both A_max_ and E eventually decreased to near-zero levels at 1600 µmol m^−2^ s^−1^. Thus, in CE plants under drought, photosynthesis and transpiration are overall reduced compared to well-watered conditions (in agreement with g_s_ reductions), but not entirely compromised. On the other hand, the nearly zeroed photosynthetic activity and transpiration of drought-stressed NB plants indicates that, in addition to stomatal conductance, some other mechanism (e.g., a biochemical mechanism) must be involved.

In both cultivars, and for both irrigation treatments, the WUE response to light (Figure 4E,F) provided additional evidence of the underlying response mechanisms to drought in the two cultivars. In WW, both cultivars achieved a maximum WUE of 4.9 µmol CO_2_ mmol^−1^ H_2_O at PPFD ≥ 900 µmol m^−2^ s^−1^, except for the drop of WUE in CE-WW at 1600 µmol m^−2^ s^−1^ driven by the reduction in A_max_ (Figure 4B). In NB, WUE was consistently lower in DS than in WW plants. On the contrary, CE plants always had the best WUE in DS plants, achieving a maximum value of 7.2 µmol CO_2_ mmol^−1^ H_2_O at PPFD = 700 µmol m^−2^ s^−1^, except for PPFD > 1400 µmol m^−2^ s^−1^. This finding confirms the theory that CE plants deal better with prolonged drought than NB plants, as this abiotic stress improves their WUE, as long as CE leaves are not exposed to excessive light, which would lead to a significant reduction in their photosynthetic capacity.

Carbon isotopic composition could be another useful method to determine drought resistance and provide insights on the chemical, physical, and metabolic processes involved in the carbon transformation of stressed plants, which, in turn, determine the water utility and water conservation status for the cultivars/species [50,51].

### 3.3. Trunk Cross-Sectional Area and Stem and Root Hydraulic Conductance

#### 3.3.1. Trunk Cross-Sectional Area

**Figure 5:**
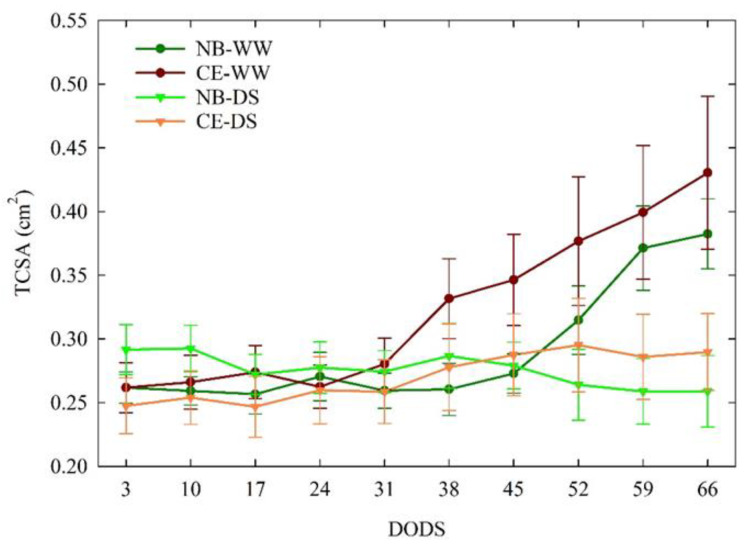
Trends of trunk cross-sectional area (TCSA) in well-watered and drought-stressed ‘Nocellara del Belice’ (NB-WW and NB-DS, respectively) and ‘Cerasuola’ (CE-WW and CE-DS, respectively) olive trees. Error bars indicate the standard errors of the means.

During the experiment, no significant differences in TCSA were detected between CE-WW and NB-WW trees (Figure 5). The TCSA of CE-WW did not show significant changes in size in the first 24 DODS and then increased almost linearly until 66 DODS, whereas in NB-WW, TCSA started to increase at 45 DODS. The TCSA of both NB-DS and CE-DS remained in the 0.24–0.30 cm^2^ range throughout the experiment. Significant differences between the TCSA of well-watered and drought-stressed plants only appeared at 59 and 66 DODS, with NB-WW and CE-WW being significantly higher than CE-DS and NB-DS.

#### 3.3.2. Hydraulic Conductance

**Figure 6:**
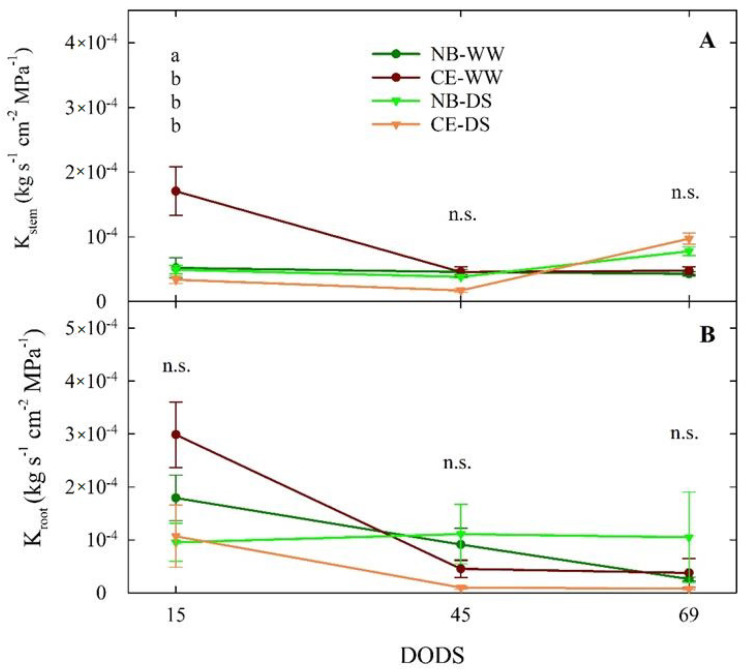
Stem sapwood−specific hydraulic conductance (K_stem_, (**A**)) and root sapwood-specific hydraulic conductance (K_root_, (**B**)) measured at 15, 45, and 69 days of drought stress (DODS) in well-watered and drought-stressed ‘Nocellara del Belice’ (NB-WW and NB-DS, respectively) and ‘Cerasuola’ (CE-WW and CE-DS, respectively) olive trees. Error bars indicate the standard errors of the means. For each day, different letters indicate significant differences between treatments according to the Tukey’s test following analysis of variance (*p* < 0.05).

Plant dissection carried out at 15 DODS revealed similar values of K_stem_ (below 1 × 10^−4^ kg s^−1^ cm^−2^ Mpa^−1^) in NB-WW, NB-DS, and CE-DS, but significantly higher hydraulic conductance in the stems of CE-WW (Figure 6A). At 45 DODS, however, the K_stem_ of all the treatment × cultivar combinations maintained similar levels below 5 × 10^−5^ kg s^−1^ cm^−2^ Mpa^−1^, which means that the stems of CE-WW significantly reduced their hydraulic conductance with time. At 69 DODS, K_stem_ remained not significantly different (*p* > 0.05) from 45 DODS, although there was a tendency for DS trees to increase their conductance compared to WW, in line with previous results showing increased K_stem_ in drought-stressed NB and CE plants [37]. This could be explained by drought-induced aquaporin regulation. Previous research has suggested that aquaporin activity is often upregulated under moderate water stress, which may in turn increase plant hydraulic conductivity [52].

Drought stress reduces plant hydraulic conductance and water status by reducing leaf water potential and sap flow. The possible reason behind this is a change in the stem anatomical structure such as xylem vessel structure, diameter, and area [53]. It is well known that an increased number of xylem vessels and an increase in their diameter has an important role on physiological functions increasing stem hydraulic conductance, sap flow, and plant photosynthetic activity [54].

The surprisingly higher value of K_stem_ measured in CE-WW at 15 DODS appeared unrelated to drought stress and may reflect the characteristics of the CE plant material used for dissection. In fact, even roots of CE-WW plants showed their highest K_root_ at 15 DODS (Figure 6B). Nevertheless, there seemed to be a decreasing trend of K_root_ over time in CE-WW, NB-WW, and CE-DS. On the other hand, K_root_ of NB-DS appeared stable around 1 × 10^−4^ kg s^−1^ cm^−2^ Mpa^−1^ from 15 to 69 DODS, which suggests that prolonged drought does not affect the conductance of NB roots or, alternatively, that the effect is immediate, and that we missed it at our first measurement after 15 DODS. The drastic reduction in K_root_ in CE-DS that was evident already at 45 DODS may have been caused either by cavitation and embolism, as demonstrated in ‘Meski’ and ‘Chemlali’ olive [27], or by loss of roots or changes in their anatomy, as observed in *Olea oleaster* by [55], or a combination. Alternatively, K_root_ responses to water deficit may be driven by reductions in the activity or concentration of aquaporins that are involved in the regulation of hydraulic conductivity [56,57,58,59,60]. Indeed, it was observed that, after 3–4 weeks under severe water stress conditions, aquaporins were significantly reduced in olive roots [61]. These findings substantiate the role of K_root_ in the adaptation of plants to different environments [62].

**Figure 7:**
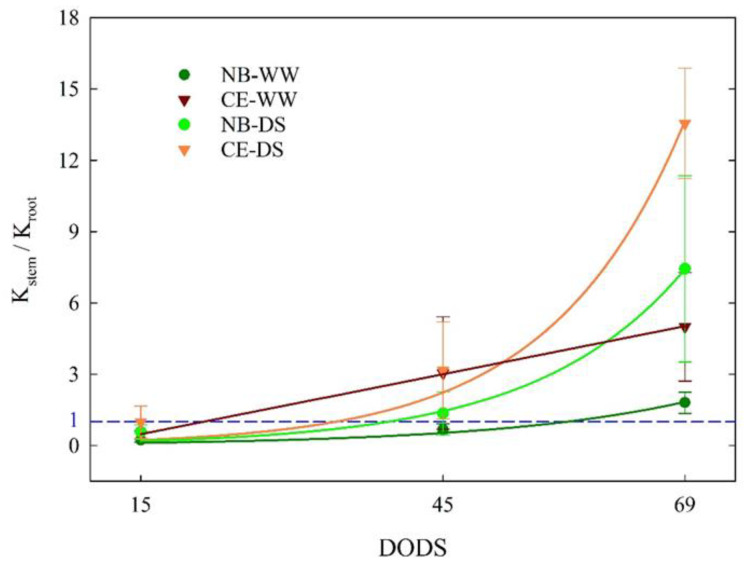
Ratio between stem sapwood-specific hydraulic conductance (K_stem_) and root sapwood-specific hydraulic conductance measured at 15, 45, and 69 days of drought stress (DODS) in well-watered and drought-stressed ‘Nocellara del Belice’ (NB-WW and NB-DS, respectively) and ‘Cerasuola’ (CE-WW and CE-DS, respectively) olive trees. Error bars indicate the standard error of the means. Curve fits interpolated using the mean values at each date. The blue horizontal line represents K_stem_ = K_root_. In NB-WW, K_stem_/K_root_ = 0.0517 × exp(0.0517 × DODS), R^2^ = 0.973; in CE-WW, K_stem_/K_root_ = −0.786 + (0.084 × DODS), R^2^ = 0.999; in NB-DS, K_stem_/K_root_ = 0.068 × exp(0.068 × DODS), R^2^ = 0.994; in CE-DS, K_stem_/K_root_ = 0.0754 × exp(0.0754 × DODS), R^2^ = 0.984.

At 15 DODS, K_stem_/K_root_ values were between 0 and 1 in all the cultivar × irrigation treatment combinations (Figure 7), indicating that, at the beginning of the drought, the highest conductance was observed in roots as opposed to stems. With time and drought intensity, K_stem_ became higher than K_root_ regardless of the cultivar × irrigation treatment combination. The increase in K_stem_/K_root_ in WW treatments might have been driven by a greenhouse environment with an increasing VPD (Figure 1). The growth of K_stem_/K_root_ from 15 to 69 DODS followed exponential trends, except for CE-WW, which followed a linear trend (Figure 7). The curvature of the exponential fits was more prominent in DS than in WW trees, with CE-DS reaching the highest ratio. These findings show that increasing the K_stem_/K_root_ ratio is an important mechanism that both cultivars, but more markedly CE, used as a drought avoidance strategy, and not only was this strategy deployed under drought conditions, but was also triggered in WW plants under warmer and drier environments such as those experienced by plants from 45 to 69 DODS.

### 3.4. Diel Dynamics of Trunk Diameter and Leaf Turgor Pressure

**Figure 8:**
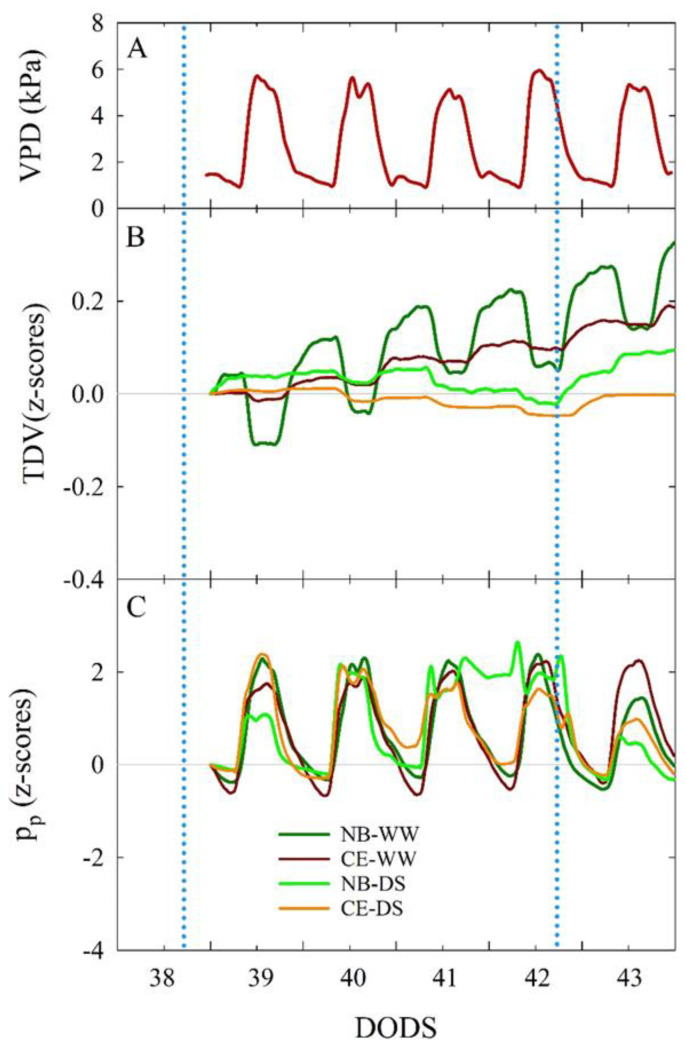
Vapor pressure deficit (VPD, (**A**)) and standardized (z-scores) of trunk diameter variations (TDV, (**B**)) and attenuated pressure of leaf patches (p_p_, (**C**)) trends in well-watered and drought-stressed ‘Nocellara del Belice’ (NB-WW and NB-DS, respectively) and ‘Cerasuola’ (CE-WW and CE-DS, respectively) olive trees. Trends reported in the time interval between 39 and 43 days of drought stress (DODS). Light blue dotted lines indicate irrigation events.

Under optimal plant water status, an inverse relationship between VPD and TDV is expected in the diurnal portion of a day [63]. This relationship was evident between 39 and 43 DODS, when TDV in both NB-WW and CE-WW decreased (Figure 8B) as VPD increased (Figure 8A) in the middle of the day. Moreover, NB-WW exhibited more marked daily fluctuations than CE-WW. The same fluctuations were nearly absent in NB-DS and CE-DS. In WW plants, TDV was mostly driven by diurnal VPD fluctuations, but also likely by the intrinsic stem growth mechanisms of young trees (i.e., expressing high relative growth rates), as suggested by the steady increase in TDV over the five days examined. Anatomical differences of xylem wood (vessel size and wall thickness and lignification) could also explain the differences between the two cultivars. The growth rates of trunk diameter between 39 and 43 DODS can be explained by comparing the slopes of linear fits of the linear regressions between TDV and DODS. Linear regressions were only significant (*p* < 0.001) for NB-WW (R^2^ = 0.544) and CE-WW (R^2^ = 0.944), as no significant stem growth was detected in DS plants between 39 and 43 DODS, confirming that shown for TCSA in Figure 7. The trunk growth rate was significantly higher in NB-WW than in CE-WW, as shown by a t-test comparison between the slopes of the linear equations (0.053 ± 0.002 and 0.038 ± 0.0004, respectively; *p* < 0.001). Variations in trunk size are correlated with changes in transpiration induced by water deficit or rehydration and have been considered more reliable than leaf water potential, photosynthetic rate, or stomatal conductance to detect initial states of water deficit in olive trees [64].

Diel dynamics of p_p_ followed a direct response to VPD in NB-WW and CE-WW, as previously observed in well-watered olive leaves [42]. This was expected as, under optimal plant water status, p_p_ is inversely related to leaf turgor, which instead decreases at high VPD due to loss of water through the stomata, driven by transpiration. The behavior of p_p_ under water deficit was described according to the classification in non-inverted, semi-inverted, and fully inverted states of p_p_ curve responses that represent no drought stress, mild drought stress, and severe drought stress, respectively, as described by [43,65]. Leaves of NB-WW and CE-WW trees were consistently in a non-inverted p_p_ state (Figure 8C). CE-DS trees entered a semi-inverted p_p_ state between 40 and 42 DODS, to then recover to no inversion after the irrigation at 42 DODS. NB-DS showed semi-inversion of the pp daily curve from 39 to 40 DODS, followed by full inversion between 41 and 42 DODS. The DS irrigation event with 30% ET at 42 DODS led to an improvement of plant water status (i.e., specifically leaf turgor) in NB-DS, as p_p_ returned to a state between no inversion and semi-inversion.

Our results indicate an improved ability of CE to maintain higher leaf turgor than NB under water deficit, although the two cultivars maintained similar g_s-morning_ (Figure 2A) in the same period. Better leaf turgor in CE may have been mostly driven by reduced cell wall elasticity [66,67], as this cultivar does not accumulate high concentrations of mannitol—a sugar alcohol that is highly active in the osmoregulation process—in its leaves [34]. Higher cell wall elasticity was thought to be one of the main factors leading to poor drought avoidance mechanisms in NB [42]. Larger and/or thinner-walled xylem vessels may also be responsible for the increased TDV diel ranges measured in NB-WW compared to CE-WW (Figure 8B).

In a previous study, CE trees appeared to reduce their leaf transpiration (expressed in terms of transpirable soil water fraction) to cope with water deficit earlier than [37]. In this study, NB was not able to detect water deficit and to trigger stress avoidance mechanisms in a timely manner when exposed to prolonged drought, which was reflected in (a) lower midday Ψ_stem_ at the end of the DS period (Figure 2B), decreased WUE (Figure 4), and fully inverted p_p_ curves (Figure 8C). However, NB-DS roots maintained similar conductance compared to NB-WW, suggesting that there was no significant increase in cavitation in the former. On the other hand, CE-DS experienced a K_root_ reduction after prolonged exposure to water deficit (Figure 8 B) that could suggest drought adaptation by reducing xylem vessels, also to avoid cavitation.

## 4. Conclusions

This study presented physiological responses and possible structural modifications in two of the main olive genotypes in the Sicilian olive germplasm—‘Nocellara del Belice’ and ‘Cerasuola’. Our findings provide empirical evidence that ‘Cerasuola’ plants express better plant water status under prolonged drought compared to ‘Nocellara del Belice’ by maintaining (i) high leaf turgor and (ii) a higher K_stem_/K_root_ ratio.

Although we provide evidence that ‘Nocellara del Belice’ trees have reduced ability to avoid drought stress, this cultivar might still be able to resume photosynthetic activity and growth even after a long drought period if soil hydration is restored. Further research on drought tolerance mechanisms in the two cultivars should be focused on measuring physiological responses and productive performance in plants that have been exposed to a period of prolonged water deficit followed by a return to optimal soil moisture conditions.

We propose that the K_stem_/K_root_ ratio can provide a good indication of drought stress avoidance mechanisms to discriminate genotype-specific strategies for coping with prolonged drought. Further research needs to be conducted to screen other olive genotypes for the presence/absence of drought avoidance mechanisms such as osmotic adjustments and reduced cell wall elasticity, and on the effect of rewatering after water deficit to estimate the drought tolerance degree in cultivars that lack drought avoidance mechanisms. The most comprehensive understanding of drought tolerance and avoidance strategies in olive cultivars can drive the selection of genotypes for resilient and sustainable olive production in environments afflicted by climate change and desertification.

## Data Availability

All data reported here is available from the authors upon request.
